# Prevalence, awareness, treatment, and control of hypertension and their determinants: Results from the first cohort of non-communicable diseases in a Kurdish settlement

**DOI:** 10.1038/s41598-019-48232-y

**Published:** 2019-08-27

**Authors:** Fatemeh Rajati, Behrooz Hamzeh, Yahya Pasdar, Roya Safari, Mehdi Moradinazar, Ebrahim Shakiba, Shahrzad Bazargan-Hejazi, Hossein Karim, Farid Najafi

**Affiliations:** 1 Research Center for Environmental Determinants of Health, Health Institute, Kermanshah University of Medical Sciences, Kermanshah, Iran; 2 Social Development & Health Promotion Research Center, Health Institute, Kermanshah University of Medical Sciences, Kermanshah, Iran; 3 College of Medicine, Department of Psychiatry, Charles Drew University of Medicine and Science & University of California at Los Angels (UCLA), Los Angels, USA; 4 Kermanshah Cardiovascular Research Center, Health Institute, Kermanshah University of Medical Sciences, Kermanshah, Iran

**Keywords:** Hypertension, Epidemiology

## Abstract

Hypertension is a public health issue in Iran. The study aimed to estimate the prevalence, awareness, treatment, and control of hypertension, and to explore their determinants among 10,040 Kurdish adults from Ravansar Non-Communicable Disease (RaNCD) cohort study in Iran. Univariate, and multivariate analyses were used for statistical analysis. Prevalence of hypertension was 15.7%. Among hypertensive patients, awareness, treatment, and control of hypertension were 80.7%, 73.2%, and 53.3%, respectively. In multivariate analysis, significant associations were found between awareness and female sex, older age, being married rather than being single, literacy, living in rural areas, having family history, and comorbidities, with a higher probability for those who had both diabetes and dyslipidemia. Being married, living in rural areas, being ex-smokers, having less physical activity and individuals who had diabetes and dyslipidemia had higher odds of receiving treatment. Being female had a statistically significant association with the control of hypertension. The Kurdish population had higher awareness, with a greater proportion of treated, and controlled patients compared to populations included in previous studies for the last 20 years in Iran. With the continuing health promotion programs in Iran, it is expected to observe a lower prevalence of hypertension, higher awareness and greater number of treated individuals with controlled hypertension.

## Introduction

Hypertension is one of the most common risk factors for cardiovascular disease (CVD)^1^. Hypertension and its complications are responsible for approximately 9.4 million deaths worldwide every year^2^. It is estimated that the the number of hypertensive patients will raise to 1.56 billion worldwide by 2025^3^. According to the report from the global burden of disease, systolic blood pressure (SBP) of at least 110 to 115 mm Hg is responsible for more than half of the global Disability-Adjusted Life Years (DALYs) in countries such as India and Indonesia^4^. World Health Organization (WHO) reported that the prevalence of raised blood pressure was 24.1% for males and 23.3% for females aged ≥18 years globally in 2014^5^.

However, almost all hypertension-related complications are preventable. Lifestyle modification helps decrease blood pressure and prevents hypertension, and antihypertensive medication can effectively reduce the cardiovascular events attributed to hypertension^6^. Adherence to medication and improving lifestyle in people with hypertension are known to decrease hospitalization, the cost associated with outpatient care, and cardiovascular mortality^7^. Moreover, improved awareness, treatment adherence, and control of hypertension in developed countries have contributed to the decline of mortality and morbidity from CVD^8^. Yet, hypertension has a silent and asymptomatic nature especially in the early stages, and therefore its detection in the community is usually delayed^9^.

The first national survey of non-communicable disease-related risk factors was initiated in 2005 in Iranian population. It showed the prevalence of hypertension among people aged 25–64 was 32%, of whom only 34% were aware of their diagnosis, with a small proportion (i.e., 25% of those who were aware) on antihypertensive treatments and a minority (i.e 24% of those receiving treatment) under control^10^. The second national survey of non-communicable disease-related risk factors reported nearly the same pattern with a small improvement, revealing that 33.3% of hypertensive individuals received treatment, of whom, 35.1% had their hypertension controlled^11^. The results of this study as well as previous studies before 2018 in Iran indicate that awareness, treatment, and control of hypertension are far from the “rule of halves”; meaning that half of the hypertensive patients are still unknown (rule 1), half of those known are not treated (rule 2), and half of those treated are not controlled (rule 3). However, we observe raised awareness among hypertensive patients and consequently an increased number of patients who have received treatment and have controlled hypertension in recently published reports^2–21^.

The Kurdish people reside in a historical area of the Middle East, which is today split between Iran, Iraq, Turkey, and Syria. Although some studies show that Kurd ethnicity has an association with a higher prevalence of hypertension^11^, this is the first community-based study on the prevalence of hypertension and risk factors related to awareness and management in a Kurdish settlement. Furthermore, the available nationally representative surveys are limited in providing information regarding risk factors and determinants of awareness, treatment, and control of hypertension^11,13,15^. The present study was designed to estimate the current prevalence of hypertension and identify factors related to its management, i.e., the level of hypertension awareness, treatment, and control. We used the baseline data of Ravansar Non-communicable Disease (RaNCD) cohort study which is the first longitudinal cohort study in a Kurdish settlement of the Middle East.

## Results

### Participants

Of the 10040 participants in the RaNCD, 25 people were deleted from the analyses due to incomplete data. From the total, 47.5% were males. 44.0% were in 35–45 age category. 24.7% were illiterate, and over 50% lived in the urban area (59.3%) (Table 1). Slightly over 20% of the participants were obese, about 12% were current smokers, and 55% reported family history of hypertension. From total, 46.9% of participants suffered from at least one of the comorbidities including diabetes (2.8%), dyslipidemia (38.7%), or both (5.3%). The prevalence of diabetes and dyslipidemia among the participants were 8.7% and 38.7%, respectively. Of the 4606 people who had a healthy diet, 17.2% were hypertensive (Table 1).

**Table 1:** Demographic and clinical characteristic of participant in RaNCD by Prevalence, Awareness, Treatment and Control.

Variables	N (%)	Prevalent cases* (95%CI)	Awareness* (95%CI)	Treatment* (95%CI)	Controlled* (95%CI)
*Total*	*10040(100%)*	1575 (15.7%)	1271(80.7%)	1153(90.7%)	840(72.9%)
**Sex**					
males	4765(47.5)	12.9(12.0,13.9)	68.0(64.2,71.6)	89.2(85.8,91.9)	65.5(60.5,70.1)
females	5275(52.5)	18.3(17.3,19.4)	88.8(86.7,90.7)	91.5(89.5,93.2)	76.4(73.3,79.3)
**Age group (year)**					
35–45	4416(44.0)	5.3(4.7,6.0)	68.0(61.7,73.7)	89.9(84.0,93.7)	80.2(72.8,86)
46–55	3339(33.3)	16.3(15,17.5)	77.7(74.0,81.0)	90.0(86.8,92.6)	71.7(67.0,76.1)
56–65	2285(22.7)	35.2(33.3,37.2)	86.5(83.9,88.7)	91.4(89.1,93.3)	72.0(68.3,75.3)
**Marital status**					
Single	420(4.2)	3.4(2,5.6)	42.9(20,69.3)	66.7(23.8,92.8)	50.0(9.5,90.6)
Married	9054(90.2)	15.8(15,16.5)	80.4(78.2,82.3)	90.7(88.8,92.2)	72.1(69.3,74.8)
Widow/divorced/other	566(5.6)	24.6(21.2,28.3)	88.5(82.1,92.9)	92.7(86.5,96.2)	80.8(72.4,87)
**Education**					
illiterate	2484(24.7)	27.9(26.1,29.7)	84.1(81.2,86.7)	91.6(89.1,93.6)	72.2(68.3,75.9)
≤5 years	3838(38.2)	14.6(13.5,15.7)	81.1(77.6,84.1)	90.1(87,92.5)	74.7(70.3,78.7)
6–9 years	1668(16.6)	9.8(8.5,11.3)	75.5(68.3,81.5)	87.0(79.8,91.9)	70.1(60.8,78.1)
10–12 years	1268(12.7)	7.5(6.1,9)	72.4(62.5,80.5)	97.1(88.9,99.3)	78.8(67.2,87.1)
≥13 years	7.79(7.8)	8.9(7.1,11.1)	68.2(56.2,78.1)	87.3(74.2,94.3)	61(45.3,74.7)
**Residential areas**					
Urban	5953(59.3)	14.9(14.0,15.8)	79.2(76.4,81.8)	87.5(84.8,89.7)	73.4(69.7,76.7)
Rural	4087(40.7)	17.0(15.9,18.2)	82.7(79.7,85.4)	94.8(92.6,96.4)	72.4(68.4,76.0)
**Economic status (n** = **9980)**					
Poorest	1996(20.0)	18.7(17.1,20.5)	81.8(77.6,85.4)	90.5(86.7,93.4)	70.7(65.0,75.8)
The 2nd poorest	1995(20.0)	17.1(15.5,18.8)	80.3(75.8,84.2)	89.4(85.2,92.6)	71.8(65.8,77.1)
Middle	1997(20.0)	16.3(14.7,18.0)	82.5(77.9,86.2)	92.9(89.2,95.5)	73.8(68.0,79.0)
The 2nd richest	2000(20.0)	14.7(13.3,16.4)	78.6(73.5,82.9)	88.8(84.0,92.3)	73.7(67.2,79.3)
Richest	1992(20.0)	11.8(10.5,13.3)	79.6(74.0,84.3)	92.6(87.8,95.6)	75.8(68.8,81.6)
**BMI (9956)**					
<18.4 (underweight)	166(1.7)	6.7(3.8,11.6)	72.8(39.9,91.5)	100(90.0–100.0)	75.0(35.2,94.4)
18.5–24.9 (normal)	2742(27.5)	10.4(9.3,11.6)	77.8(72.6,82.3)	91(86.4,94.1)	76.5(70.2,81.9)
25.0–29.9 (overweight)	4335(43.5)	15.1(14.1,16.2)	81.8(78.6,84.6)	91.2(88.5,93.4)	70.8(66.6,74.7)
30.0–34.9 (class I obesity)	2130(21.4)	21.6(19.9,23.4)	81.3(77.5,84.6)	90.7(87.3,93.2)	75.2(70.3,79.5)
≥35 (class II obesity)	583(5.9)	25.8(22.4,29.5)	80.7(73.6,86.3)	88.5(81.4,93.1)	68.3(58.8,76.4)
**WHR(n** = **9962)**					
Normal	1762(17.6)	9.6(8.3,11.0)	74.3(67.1,80.4)	88.8(81.8,93.3)	71(61.8,78.7)
Central obesity	8220(82.4)	17.0(16.2,17.8)	81.5(79.4,83.5)	91.0(89.2,92.6)	73.1(70.3,75.8)
**Smoking (n** = **10015)**					
Non smoker	8011(80.0)	15.7(14.9,16.5)	82(79.8,84.1)	90.7(88.7,92.3)	72.7(69.7,75.5)
Current = Lifetime	1178(11.8)	11(9.3,12.9)	76.8(68.7,83.3)	86.9(78.7,92.3)	78.0(67.9,85.5)
ex-smoker	826(8.2)	23.4(20.7,26.4)	75.2(68.6,80.8)	94.5(89.4,97.3)	71.6(63.4,78.5)
**Physical activity Daily METs**					
24–36.5 = Low	2765(27.6)	18.2(16.8,19.7)	83.7(80.3,86.7)	92.0(89.0,94.2)	75.0(70.4,79.1)
36.6–44.9 = moderate	5158(51.4)	15.9(14.9,16.9)	82.6(79.8,85.0)	91.0(88.6,93.0)	72.4(68.7,75.8)
≥45 = vigorous	2109(21.0)	12.1(10.8,13.6)	68.9(63.0,74.4)	86.9(81.0,91.2)	69.8(62.0,76.6)
**Family history of hypertension(n** = **10022)**					
No	4533(45.2)	12.5(11.6,13.5)	72.1(68.2,75.6)	88.7(85.3,91.5)	70.4(65.5,74.9)
Second degree	5002(49.9)	17.5(14.4,21.1)	71.8(61.3,80.4)	83.7(72.1,91)	72.6(58.7,83.2)
First degree	487(4.9)	18.5(17.5,19.6)	87(84.7,89.1)	92.3(90.3,94)	74.1(70.9,77.2)
**Comorbidities**					
None	5332(53.1)	12.1(11.3,13)	79.0(75.6,81.9)	88.9(85.8,91.3)	71.7(67.4,75.7)
Diabetes	280(2.79)	30(25,35.7)	87.0(77.9,92.7)	91.8(82.8,96.3)	74.7(62.9,83.7)
Dyslipidemia	3889(38.7)	16.7(15.6,17.9)	79.2(75.9,82.2)	91.1(88.3,93.3)	73.9(69.7,77.7)
Both	539(5.37)	36.8(32.8,40.9)	88.9(83.7,92.6)	94.9(90.5,97.4)	72.5(65.2,78.8)
**Healthy diet**					
Yes	4606(45.8)	17.2(16.1,18.3)	82.2(79.3,84.7)	90.0(87.4,92.1)	74.3(70.6,77.7)
No	5434(54.1)	14.5(13.6,15.5)	79.3(76.4,82)	91.6(89.1,93.5)	71.5(67.7,75.1)

*Proportion in each column has been calculated using the values in previous column as a denominator.

### Prevalence, awareness, treatment, and control of hypertension

**Figure 1:**
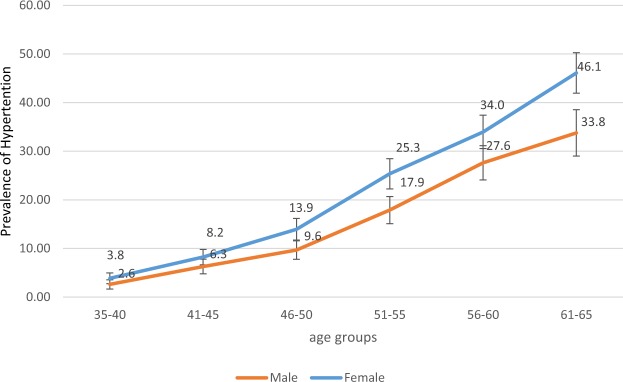
The prevalence of hypertension by age and sex.

Nearly 16% of the overall study population were hypertensive (15.7% [CI = 15.0–16.4] of whom the highest proportion (35.2%) was in 56–65 age category (Table 1). The mean systolic BP of the population was 108.5 ± 17.6 (SD) mmHg and mean diastolic BP was 69.8 ± 10.2 (SD) mmHg. BP increased with age p < 0.001 (Fig. 1). In all age groups, the prevalence of hypertension in women was higher than that of men p < 0.001 (Fig. 1). However, the difference was statistically significant only after 45 years of age (p < 0.05).

Of the 1575 (15.7%) hypertensive participants, 1271 (80.7%) were aware of their condition, 1153 (73.2%) received treatment, and 840 (53.3%) had controlled blood pressure. Among those who were aware of their hypertension status, the percentage who had received antihypertensive treatment was 90.7%. Yet, there were 304 (19.3%) and 735 (46.7%) participants who were not aware of their hypertensive condition and had uncontrolled blood pressure, respectively.

As presented in Table 2, of the participants who were hypertensive and were taking medication, 90 individuals (7.8%) were in stage 2 (systolic pressure of 140 mm Hg or higher or a diastolic pressure of 90 mm Hg or higher).

**Table 2:** Univariate and multivariate analyses of factors affecting prevalence, awareness, treatment and controlled of hypertension in RaNCD. *Adjusted for all variables cited in the table.

Variables			Prevalence		Awareness among hypertensive patients		Treatment among aware patients		Control among treated patients	
			Crude OR(95%CI)	*Adjusted OR(95%CI)	Crude OR(95%CI)	*Adjusted OR(95%CI)	Crude OR(95%CI)	*Adjusted OR(95%CI)	Crude OR(95%CI)	*Adjusted OR(95%CI)
sex	(Ref: male)		1.6(1.4,1.7)	1.1(1.0,1.3)	3.7(2.9, 4.9)	4.9(3.4,7)	1.4(0.9,2.0)		1.8(1.4,2.3)	1.7(1.3,2.3)
Age	(Ref: 35–45)	46–55	3.6(3.0,4.2)	3.2(2.7,3.8)	1.7(1.2,2.4)	1.9(1.3,2.8)	1.1(0.6,1.9)		0.7(0.4,1.1)	0.7(0.5,1.1)
		56–65	9.9(8.4,11.5)	8.7(7.2,10.5)	3.1(2.2,4.3)	4.3(2.8,6.7)	1.2(0.7,2.2)		0.7(0.5,1)	0.7(0.5,1.1)
Marital status	(Ref: single)	Married	5.5(3.2,9.3)	2.4(1.4,4.1)	5.5(1.9,15.8)	8.1(2.5,27.1)	4.9(0.9,26.8)	15.6(2.7,92)	2.6(0.4,18.5)	
		Widow/divorced/other	9.5(5.4,16.7)	2.1(1.2,3.8)	10.3(3.2,33.4)	6.9(1.9,26)	6.4(1.1,39.5)	18.5(2.8,122.6)	4.2(0.6,31.4)	
Education	(Ref: illiterate)	≤5 years	0.5(0.4,0.6)	0.8(0.7,1)	0.9(0.7,1.1)	1.4(1,2)	0.9(0.6,1.3)		1.2(0.9,1.6)	
		6–9 years	0.3(0.3,0.4)	0.7(0.6,0.9)	0.6(0.4,0.9)	2(1.2,3.3)	0.7(0.4,1.2)		1(0.6,1.5)	
		10–12 years	0.3(0.2,0.3)	0.7(0.5,0.9)	0.5(0.4,0.9)	2.1(1.2,4)	3.1(0.8,12.8)		1.5(0.8,2.7)	
		≥13 years	0.3(0.2,0.4)	0.8(0.5,1.1)	0.5(0.3,0.7)	1.7(0.9,3.4)	0.7(0.3,1.6)		0.7(0.4,1.2)	
Residential area	(Ref: Urban)		1.2(1.1,1.4)	1.4(1.3,1.6)	1.3(1,1.7)	1.6(1.2,2.1)	2.7(1.7,4.1)	3.5(2.2,5.5)	1.1(0.8,1.3)	
Economic status	(Ref: Poorest)	2nd poorest	0.9(0.8,1.1)	1.0(0.8,1.2)	1.0(0.7,1.4)		0.9(0.6,1.6)		1.1(0.73, 1.55)	
		Middle	0.9(0.8,1.0)	1.1(0.9,1.3)	1.1(0.8,1.6)		1.4(0.8,2.6)		1.2 (0.8,1.7)	
		2nd richest	0.8(0.7,0.9)	1(0.8,1.2)	0.9(0.6,1.2)		0.9(0.5,1.5)		1.2(0.8,1.8)	
		Richest	0.6(0.5,0.7)	1(0.8,1.2)	0.9(0.6,1.4)		1.3(0.7,2.6)		1.3(0.9,2.0)	
BMI	(Ref: 18.5–24.9)	18.4≤	1.7(0.9,3.1)	1.7(0.9,3.2)	1.4(0.4,5.1)		*		1.09(0.2, 5.6)	
		25.0–29.9	2.5(1.4,4.7)	2.4(1.3,4.7)	1.7(0.5,6.5)		1.0(0.6,1.8)		0.81(0.17,4.05)	
		30.0–34.9	3.9(2.1,7.2)	3.7(1.9,7.3)	1.7(0.5,6.3)		0.9(0.5,1.7)		1.1(0.2,5.1)	
		≥35	4.9(2.6,9.3)	4.9(2.5,9.8)	1.6(0.4,6.3)		0.7(0.4,1.5)		0.8(0.2,3.8)	
WHR	(Ref: Normal)		2.0(1.7,2.3)	1.1(0.9,1.3)	1.6(1.1,2.3)	0.8(0.5,1.2)	1.3(0.8,2.4)		1.12(0.7,1.7)	
Smoke Cigarette	(Ref: Non-smoker)	Current	0.7(0.6,0.9)	0.8(0.7,1.0)	0.8(0.5,1.2)		0.7(0.4,1.3)	0.7(0.4,1.3)	1.4(0.8,2.3)	
		ex-smoker	1.7(1.4,2.0)	1.2(1,1.4)	0.7(0.5,1.0)		1.8(0.9,3.8)	2.2(1,4.7)	1(0.7,1.5)	
Physical activity	(Ref:24–36.5)	36.6–44.9	0.9(0.8,1)	0.9(0.8,1)	1.0(0.7,1.3)	0.8(0.6,1.1)	0.9(0.6,1.4)	0.9(0.6,1.4)	0.9(0.7,1.2)	
Daily MET		≥45	0.7(0.6,0.8)	0.8(0.7,0.9)	0.5(0.4,0.7)	0.7(0.5,1.1)	0.6(0.4,1.1)	0.5(0.3,0.9)	0.8(0.6,1.2)	
Family history of hypertension	(Ref: No)	Second degree	1.5(1.2,2)	1.3(1.1,1.7)	1.0(0.6,1.7)	1.2(0.7,2)	0.7(0.4,1.4)	0.6(0.3,1.2)	1.2(0.6,2.2)	
		First degree	1.6(1.5,1.8)	2.2(1.9,2.4)	2.6(2.0,3.4)	2.9(2.2,3.9)	1.6(1.1,2.3)	1.7(1.1,2.5)	1.3(1,1.6)	
Comorbidities	(Ref: None)	Diabetes	3.2(2.4,4.1)	2.2(1.7,3)	1.8(1.0,3.5)	1.9(0.9,3.8)	1.5(0.6,3.4)	1.7(0.7,4.2)	1.2(0.7,2.1)	
		Dyslipidemia	1.5(1.3,1.7)	1.4(1.2,1.6)	1.1(0.8,1.4)	1.2(0.9,1.7)	1.3(0.9,2.0)	1.4(0.9,2.1)	1.2(0.9,1.5)	
		Both	4.3(3.5,5.2)	2.7(2.2,3.3)	2.2(1.4,3.5)	2.5(1.5,4.3)	2.4(1.2,4.9)	3.0(1.4,6.3)	1.1(0.7,1.6)	
Healthy diet	(Ref: Yes)	No	0.9(0.8,1)	0.9(0.8,1)	0.9(0.7,1.1)		1.3(0.9,1.8)		0.9(0.7,1.2)	

### Determinates of prevalence, awareness, treatment, and control of hypertension

In the bivariate analysis, we found association between an increased risk of hypertension and the following factors: being female, older age, married, a widow/divorced, and ex-smoker, living in a rural area, and having higher BMI, central obesity, diabetes and dyslipidemia and family history of hypertension. On the other hand, a higher level of physical activity and education was related to a lower risk of hypertension. (Table 2). Overall, those who had 10–12 years of education were less likely to be at risk of hypertension compared to their counterparts [OR (95% CI), 0.61(0.47–0.81)]. However, living in a rural area and obesity of class I and class ≥II increased the odds of having hypertension by 1.4(1.3–1.6), 3.7(1.9–7.3), and 4.9(2.5–9.8), respectively. Current smokers in the crude and adjusted model had a lower risk of hypertension than ex-smokers. In addition, the likelihood of having hypertension increased with diabetes [OR (95% CI), 2.2(1.7–3.0)], dyslipidemia [OR (95% CI), 1.4(1.2–1.6)], having both of them [OR (95% CI), 2.7(2.2–3.3)] and lower values for Metabolic Equivalent Tasks (METs). Compared with the participants without any family history of hypertension, odds of being hypertensive was 1.3 for those who had a positive history of hypertension in the second-degree relatives, with 2.2 in their immediate family (Table 2). In both unadjusted and adjusted models, women were more likely to be aware of their hypertension status, [AOR (95% CI), 4.9 (3.0–4.7)], and the likelihood of awareness was higher in the older age category compared with their younger counterparts [AOR (95% CI), 4.3(2.8–6.7)]. On the other hand, rural people compared to those living in urban areas [AOR (95% CI), 1.6(1.2–2.1)], and people with a family history of hypertension in their immediate family compared to those without a positive family history [AOR (95% CI), 2.9(2.2–3.9)] had higher likelihood of being aware of their hypertension condition in both unadjusted and adjusted models. Participants with normal WHR were more likely to be aware of hypertension in adjusted model. In addition, diabetics [OR (95% CI), 1.9(0.9–3.8)] and individuals with dyslipidemia [AOR (95% CI),1.2(0.9–1.7)] had higher odds of awareness. Crude and adjusted model showed a statistical association between awareness regarding hypertension and comorbidity of having both diabetes and dyslipidemia [AOR (95% CI), 2.5(1.5–4.3)] (Table 2). Before and after adjustment, there was no association between sex and treatment. Compared to single participants, those who were married were more likely to receive treatment [AOR (95% CI)], 15.5 (2.7–92.0). The odds of treatment in rural people was higher than urban people even after adjustment [AOR = 3.5; CI (2.2–5.5)]. While ex-smokers compared to non-smokers had higher likelihood of receiving treatment for hypertension in multivariable model, those who had higher METs (≥45) compared to lower METs (24–36.5) were less likely to receive treatment [AOR (95% CI), 0.6(0.4–1.1)]. In addition, patients with positive history of hypertension in their immediate family had greater likelihood of being treated [(AOR (95% CI), 1.6(1.1–2.3)]. Participants with both diabetes and dyslipidemia were more likely to get treatment for hypertension than those who had diabetes or dyslipidemia alone. Patients who were not on a healthy diet had more likelihood of getting treatment [(AOR (95% CI), 1.3 (0.9–1.8) (Table 2).

The probability of controlling hypertension was higher among women [AOR (95% CI), 1.7 (1.3–2.3)]. The univariate model showed a significant association between control of hypertension and having a positive history in the immediate family [OR (95% CI), 1.3(1.1–1.6)]. People with higher wealth index, education central obesity, and those with comorbidities had a marginally higher likelihood of control of hypertension in the unvitiated analysis. However, in the adjusted analysis, the above mentioned variables lost their significance. The adjusted model showed that ex-smokers compared to non-smokers were more likely to receive treatment for their hypertension [OR (95% CI), 2.2(1–4.7)]. Although BMI was not associated with control of hypertension, lower BMI could increase the odds of hypertension control by 5.56 (Table 2).

## Discussion

Our findings revealed that nearly 16% of our adult population in the study had hypertension, with the highest percentage in 56–65 age category. The prevalence of hypertension in Iran shows a great geographical variation, ranging from 4.5% in Ghazvin to 46.9 in Tehran^22^. While “the rule of haves” suggests that almost 50% of hypertensive patients are aware of their disease^23^, in the case of our hypertensive patients, 80.0% were aware of their hypertensive status, of whom 90.7% of those who were aware received treatment for their hypertension, and of whom 72.8% had controlled Although these findings suggest considerable improvement in awareness and access to treatment and control, approximately one out of four treated hypertensive individuals (28%) was found to have uncontrolled hypertension. In addition, 20% of people who were not aware of their hypertension and 9.3% of those who were aware of their hypertension did not receive any treatment, which is alarming for the Iranian health care system due to its aging population. In fact, not only physicians and health care providers but also health policy makers need to reemphasize five levels of prevention including primordial, primary, secondary, tertiary, and quaternary prevention. While the reason for lack of control among 28% of treated hypertensive patients is not exactly understood, it is necessary to focus on all strategies embedded in the five levels of prevention to control hypertension. These strategies include political, social, and cultural interventions(i.e. changing unhealthy lifestyle; conducting mass, targeted or opportunistic screening; empowering patients to self-manage their hypertension)^24^. All these findings are in line with a recently published systematic review and meta-analysis on this issue in Iran^25^.

**Figure 2:**
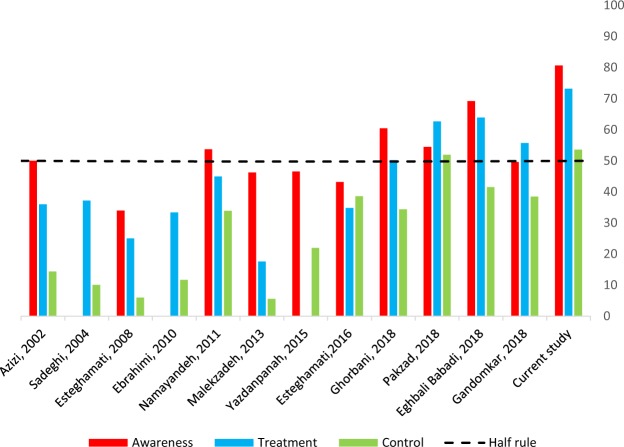
Awareness, treatment and control of hypertension among hypertensive adults.

To provide a better comparison between studies that have investigated the prevalence, awareness, treatment and control of hypertension at both national and regional levels among Iranian adults, a visual summary of study results is depicted in Fig. 2. As it can be seen, an upward trend is obvious in terms of awareness, treatment, and control of hypertension among Iranian people from 2000 to 2018. While the prevalence of hypertension in our study was lower in comparison to other studies, the awareness, treatment, and control have shown improvement (Fig. 2). Previous reports also confirmed the rising trend over time^26,27^.

Figure 2 also shows that hypertension prevalence ranges from 17.0% in a study by Sadeghi *et al*. (2004) to 42.7% in Golestan cohort study. Regardless of geographical variations, the major difference in the prevalence of hypertension in these studies is in the screening methods. In the current study, BP on each arm was measured twice during the screening, whereas in the surveillance of risk factors of Non-Communicable Diseases studies, authors measured blood pressure three times sequentially in one visit^17^. Also, studies with older participants have reported a higher prevalence of hypertension. For example, Golestan cohort study included people aged 40–75 and reported hypertension prevalence of 46.7% vs. Ebrahimi *et al*. (2010) included individuals aged 15–64 with 17.4% prevalence1^1,15^. Although hypertension awareness in published studies before 2018 was less than 50%, it ranged from 49.6% to 69.2% in four published surveys in 2018^18–21^. These figures are lower than our finding of 80.7%. With the exception of Golestan cohort study and Surveillance of Risk Factors of Non-Communicable Diseases-2005, all studies reported that more than 25.0% of hypertensive patients received antihypertensive medication. We demonstrated the highest proportion of medication consumption in all patients at 73.2%. Compared to these studies, we observed the highest control rate of hypertension in our study at 53.3%.

While in a study of hypertensive Polish patients, up to 82% was found to receive treatment, 41% of their Indian counterparts received such treatment in another study^28,29^. Taking these findings into account, one can argue that our hypertensive population has passed “the rule of halves”. This indicates that Iran has been successful in initiating several health reforms and introducing a nationwide network of health care facilities to provide and disseminate public health programs to the country. The hypertension-related gaps between Iran and low to middle-income countries is currently narrower^30^, and our estimate competes with countries such as Great Britain^31^. Indeed, since 2014, Iran has implemented the Package of Essential Non-communicable Disease (IraPEN), which is the Iranian adapted version of WHO’s Package of Essential NCD^32^. Moreover, Iran’s newly instituted nationwide health insurance program has helped raise public awareness of hypertension and control it in Ravansar^31^. Similar results indicate a relationship between insurance coverage and control rates of blood pressure^33^. A study conducted on different ethnicities confirmed the association between Kurdish ethnicity and poor psychological well-being^34^. Such poor well-being may result in higher chances of seeking medical and health care and consequently higher patients’ awareness and treatment. Moreover, the success in awareness-raising on hypertension and its control in Ravansar is partly due to the national campaigns implemented across the country every year.

A key finding of the present study was a higher prevalence of hypertension among women in all age groups. While 1 in 4 women aged 45–50 suffered from hypertension, the corresponding value for men was 1 in 5. A recent review on the gender difference in hypertension indicates that hypertension prevalence is higher in men than women until after menopause^35^. There is well-documented evidence that estrogen protects women from the earlier occurrence of hypertension^36^.

A study conducted in 17 countries in individuals aged 35 to 70 with different income levels showed that men (including Iranians) had totally a higher prevalence of hypertension than women with the exception of low-income countries^37^. In our study, women were more likely to suffer from hypertension. Women in RaNCD cohort study were more overweight and obese (46.64% vs. 40.73% for overweight and 36.55% vs. 16.86% for obese women and men, respectively). Previous studies showed Kurdish women were more likely to be overweight and obese than women of other ethnicities^38^. Also, women were more likely to be aware of their hypertension, and they were more likely to have their hypertension controlled compared to men. Such findings correspond with previous national studies in Iran^10^ and findings from other countries^39^. The difference may be explained by higher level of attention to the health care and adherence to the prescribed medications among women^40^. There is some evidence that males in younger age groups have higher systolic and diastolic BP than females. These values increase in females across older age groups^10^. However, results from some Iranian studies show that this pattern might remain unchanged during older ages for hypertensive patients^12^. Similar to results from China, in the current study, we found no significant difference between men and women in terms of values for both systolic and diastolic BP^41^.

In the present study, awareness increased with aging. Literature shows inconsistency in relationship between age and awareness, treatment and control of hypertension^42^. Moon and *et al*. (2013) found a positive association between the control of hypertension and aging in people aged 65 and over and also in younger age groups^43^. Aging is often positively associated with the awareness of some chronic condition such as diabetes and hypertension^41,44^. This might be due to gradual raised awareness brought about by aging.

There was a difference in odds of prevalence of hypertension, awareness, treatment, but not in control between married and other groups. The reverse results have been reported on the relationship between marriage and prevalence of hypertension^45^. As we expected that family support positively affects the adherence to prescribed medication in hypertensive patients^46^, the RaNCD also showed a significant association.

Also, prevalence of hypertension in all educated groups was lower compared to illiterates. Awareness of hypertension was statistically associated with the level of education with the exception of higher-educated people who were not significantly different from other groups. However, treatment and control of the hypertension were not associated with the education (Table 2). This could be due to the nationwide coverage of primary health care which provides access to health care for all. It is in line with other studies conducted in Iran that reported individuals with higher education used less health care services^47^. This means that higher-educated people are less likely to receive health care. Accordingly, no association was found between prevalence of hypertension, awareness, and treatment with wealth index.

While the previous studies showed that prevalence of hypertension was not related to living area, in the current study prevalence of hypertension, awareness, and treatment were positively associated with living in rural area^10,11^. This may be explained by the fact that people in rural areas have the advantage of access to primary health care provided by health workers called Behvarz. Through Health Houses established a few decades ago^48^, this network has offered different NCDs related services including diabetes, hypertension and mental disorders over the years. While there is an active surveillance for conditions such as hypertension and diabetes in rural areas, urban people will be followed only if they visit a public health center^46,49^. Yet, our findings are not in line with studies from elsewhere^50^.

The current study found that there is a positive association between body mass index and hypertension. Greater BMI is correlated with higher levels of fat mass, an increase in salt retention and insulin resistance^51^ which results in an increased high blood pressure. Similar to previous studies, we observed a positive association between awareness of hypertension and central obesity in univariate model^12,15^. Awareness, treatment and control of hypertension were not related to BMI after adjustment.

We found that former smokers were more likely to have hypertension. However, current smoking was the significant protective factor for hypertension in univariate analysis. Counter to the evidence that shows smoking increases the risk of hypertension^52,53^, the causal relationship between smoking and hypertension has been well documented for the “incident” cases of hypertension^54^. The evidence shows the relationship between smoking and hypertension is more likely related to the length of smoking and lifetime cigarette consumption rather than being a current smoker^55^. In addition, similar to our study, some epidemiological research^56^ reported that current smokers have lower blood pressures compared to non-smokers and even ex-smokers. In fact, in our study, although smokers had higher risk of higher prevalence of hypertension than non-smokers, this likelihood is including null in multivariable analyses even with such a large sample size. Such attenuation of effect estimate is largely due to the confounding effect of BMI. In fact, smokers have lower prevalence of being overweight and obese (53.3%) compared to non-smokers (66.6%) and ex-smokers (64.75%).This may be related to reverse causation, meaning that prevalent cases of hypertension have quit smoking after diagnosis. Also, in our study, being an ex-smoker was highly correlated with receiving antihypertensive treatment. Quitting smoking and getting treatment for cardiovascular events were also associated with each other after myocardial infarction as expected^57^.

As for the physical activity, its lower level was associated with higher prevalence of hypertension. This is in line with literature saying that the absence of moderate to vigorous physical activity is associated with an increased risk of hypertension^58^. Yet surprisingly, the group with the highest level of physical activity had less likelihood of being aware of their hypertension and receiving medication.

Individuals with a history of hypertension in their immediate family were more likely to have hypertension than those who had such history in their second-degree relatives. Patients with family history of hypertension in their immediate family were also more aware of their condition with higher probability of receiving treatment (Table 2). This is in line with the result of zhang’s *et al*. (2017), reporting that hypertensive people with a family history of coronary heart diseases were more likely to have controlled hypertension^59^.

In our study, patients who had diabetes and/or dyslipidemia had higher probability of being hypertensive, aware, and treated. Often patients with comorbid disease have higher perception of the risk factors and their condition. Hypertension is a common comorbid condition with diabetes and dyslipidemia. A higher rate of hypertension treatment in diabetic patients has been reported in literature^60^. The odds of awareness and treatment is also higher in diabetic patients. Our finding emphasizes the importance of regular patients screening. A healthy diet also correlated with prevalence of hypertension, suggesting that individuals tend to change their diet after incidence of hypertension.

The current study had four limitations. Firstly, due to its cross-sectional design, exploring the casualty was limited. Secondly, our data included the Kurdish people living in western part of Iran which limits the generalizability of the result to the nationwide. However, Iranian health network has a national coverage which narrows the disparity in access to care and provides equitable services. Thirdly, patients’ previous experience of medications for other conditions could have contributed to their current adherence to treatment. Further studies are needed to evaluate the correlation between patients’ previous experience of taking medication and current hypertension treatment. Lastly, we did not investigate the effect of cognitive factors such as self-efficacy and illness perception in people who received treatment and had controlled hypertension.

In conclusion, our findings show a higher level of awareness, treatment, and control of hypertension in the study population compared to similar findings from recently published Iranian studies in the last two decades. Future studies are needed to shed more light on hypertension-related outcomes of the ongoing health promotion programs in Iran (such as IraPEN).

## Methods

### Study design and population

The RaNCD cohort study is a part of the Prospective Epidemiological Research Studies of Iranian Adult (PERSIAN) Cohort that focuses on permanent residents of Ravansar aged 35–65 years. In the PERSIAN cohort, all 19 cohort sites (covering a representative sample of different Iranian ethnicities) used the same questionnaire and aimed to follow up all participants for the next 15 years. Further information is available at http://persiancohort.com.

Ravansar, is a district of Kermanshah province located in the western part of Iran. It is a mountaneous area with moderate climate and a population of 47657. Most of the residents belong to Kurdish ethnicity and speak Kurdish. Approximately, 15000 people aged 35–65 live in both urban and rural areas of Ravansar. Using census sampling, a total of 10065 people were recuited based on all available resources and signed informed consent. The response rate was93.3%^61^. Participants were recruited between November 2014 and February 2017. The details of this study have already been published^61^. For the purpose of this study, we used the baseline data of RaNCD. The study was approved by the ethics committees of Kermanshah University of Medical Sciences (KUMS.REC.1394.315), Kermanshah, Iran. Every participant in the study provided a written informed consent.

### Data collection and measurements

Trained personnels with good command of Kudish conducted all interviews and obtained all the measurements through paying homevisits for all the participants. All selected participants received a reminder call one day in advance of their scheduled appointment. Participants were also advised to be fasting on the day of appointment^62^.

The questionnaires consisted of several domains including socio-demographics, medical history, clinical data/biomarkers, lifestyle, and history of previous and current medication for hypertension and diabetes. It took approximately one hour to complete the questionnaire for each participant.Wealth index defined based on the selected assets including home ownership, area per capita, room per capita, having freezer, laundry machine, dish washer, personal computer, access to internet, motorcycle, vacuum cleaner, TV at the household and having cellphone, laptop access to internet, and car(based on its price) for personal use. Principal component analysis (PCA) was applied to asset data to create wealth index, assigning a score for each variable and then adding them up to determine wealth score. Participants were ranked and then divided into five quintiles including poorest, second poor, middle, second rich and richest, according to their total asset score.

To measure weight, we used a Bio Impedance Analyzer BIA (InBody 770 BIOSPACE, KOREA). Height was measured with 0.1 cm accuracy with stadiometer. We calculated the participants’ body mass index (BMI) by dividing weight (kg) by height (m^2^) and subsequently grouped them into different weight categories. We categorized Individuals with a BMI less than 18.5 kg/m^2^ as underweight, between 18.5–24.9 Kg/m^2^ as normal, between 25.0 and 29.9 kg/m as overweight, between 30 and 34.9 as obese Class I, and greater than 35 as obese Class II or III. We calculated waist to hip ratio (WHR) by dividing waist circumference to hip circumference. The value of central obesity, which is measured based on WHR, was defined as 0.90 and greater than or equal to 0.85 for men and women, respectively. The participants smoking status was evaluated based on the National Health Interview Survey (NHIS)^63^. The 24-hour physical activity was measured by asking participants about their sport, work, and leisure- related activities on an average weekday, which then grouped them into different categories^64^ (Table 1). Dyslipidemia was defined as LDL cholesterol (mg/dL) ≥ 160 and/or total cholesterol (mg/dl) ≥ 240 and/or HDL cholesterol (mg/dL) < 40 and/or triglycerides (mg/dL) ≥ 200 and/or having a history of medication for this condition. Total cholesterol was measured through enzymatic method. Triglyceride, LDL-cholesterol, and HDL-cholesterol levels were measured by colorimetric method.

Diabetes mellitus was defined as Fasting Blood Sugar (FBS) >=126 mg per dL [7 mmol per L] and/or history of medication for diabetes treatment (insulin and/or oral hypoglycemic agents)^31^. Healthy diet was defined as eating at least 400 gram fruits and vegetable per day, less than 30 percent of daily energy intake of fat, less than 10 percent daily energy of sugar and less than 5 grams salt per day^65^.

Sitting blood pressure was measured through a standardized procedure after 10 minutes of rest with two measurements of right arm and two measurement of left arm with cuff size adjusted to arm circumference. The cuff was placed on the arm at the level of the heart using a Riester duplex blood pressure. There was at least one minute interval between two separate measurements. The average of two measurements for each arm was calculated. The higher measurement of two arms was considered as mean of systolic and diastolic blood pressure (DBP).We used the Joint National Committee on Prevention, Detection, Evaluation, and Treatment of High blood pressure (JNC-7) classification of hypertension to diagnose the hypertension^6^. So, this study considered participants with a SBP ≥ 140 mm Hg and/or DBP ≥ 90 m Hg and/or those with a current use of antihypertensive drugs as hypertensive. Individuals who had systolic BP = 140–159 mm Hg and/or diastolic BP = 90–99 mm Hg were categorized as having stage 1 hypertension. In addition, SBP ≥ 160 mm Hg and/or diastolic BP ≥ 100 mm Hg was defined as stage 2 hypertension. We used self-report of physician-diagnosed hypertension to measure participant’s awareness of this condition. In addition, those who received prescribed blood pressure medications were classified as treated and if they had systolic blood pressure of <140 mm Hg and a diastolic blood pressure of <90 mm Hg they were classified as controlled hypertension. All methods were performed according to the relevant guidelines^6^.

### Statistical analysis and calculations

We used descriptive statistics to report the population characteristics and examine the distribution of the variables. Continuous variables were reported as mean and standard deviation (SD), and categorical variables were reported as number and percent. We tested the difference in the systolic and diastolic blood pressure by gender using t-test, and tested the bivariate association between categorical variables and study outcome variable i.e., hypertension, level of awareness, treatment, and control using chi-square test. For such tests, the significance level was less than 0.05. Subsequently, multiple regression model was adjusted for all variables with a *p*-value below 0.3 in univariate analysis. We used adjusted logistic regression via the enter method to assess their independent relations with the outcome variables. Data were presented as adjusted odds ratio (OR) and 95% confidence intervals (CI). Data were analyzed using Stata software (version 14.1) (Stata Corp, College Station, TX, USA).

## Data Availability

The data and the detail of all analyses will be available on request.
